# Evaluation of receptor‐ligand mechanisms of dual‐targeted particles to an inflamed endothelium

**DOI:** 10.1002/btm2.10008

**Published:** 2016-06-20

**Authors:** Catherine A. Fromen, Margaret B. Fish, Anthony Zimmerman, Reheman Adili, Michael Holinstat, Omolola Eniola‐Adefeso

**Affiliations:** ^1^ Dept. of Chemical Engineering University of Michigan Ann Arbor MI 48109; ^2^ Dept. of Pharmacology University of Michigan Ann Arbor MI 48109; ^3^ Division of Cardiovascular Medicine Samuel and Jean Frankel Cardiovascular Center, University of Michigan Ann Arbor MI 48109

**Keywords:** dual‐targeted particle, intravital microscopy, leukomimietics, ligand‐receptor pair, particle adhesion, vascular‐targeted carrier

## Abstract

Vascular‐targeted carriers (VTCs) are designed as leukocyte mimics, decorated with ligands that target leukocyte adhesion molecules (LAMs) and facilitate adhesion to diseased endothelium. VTCs require different design considerations than other targeted particle therapies; adhesion of VTCs in regions with dynamic blood flow requires multiple ligand‐receptor (LR) pairs that provide particle adhesion and disease specificity. Despite the ultimate goal of leukocyte mimicry, the specificity of multiple LAM‐targeted VTCs remains poorly understood, especially in physiological environments. Here, we investigate particle binding to an inflamed mesentery via intravital microscopy using a series of particles with well‐controlled ligand properties. We find that the total number of sites of a single ligand can drive particle adhesion to the endothelium, however, combining ligands that target multiple LR pairs provides a more effective approach. Combining sites of sialyl Lewis A (sLe^A^) and anti‐intercellular adhesion molecule‐1 (aICAM), two adhesive molecules, resulted in ∼3–7‐fold increase of adherent particles at the endothelium over single‐ligand particles. At a constant total ligand density, a particle with a ratio of 75% sLe^A^: 25% aICAM resulted in more than 3‐fold increase over all over other ligand ratios tested in our in vivo model. Combined with in vivo and *in silico* data, we find the best dual‐ligand design of a particle is heavily dependent on the surface expression of the endothelial cells, producing superior adhesion with more particle ligand for the lesser‐expressed receptor. These results establish the importance of considering LR‐kinetics in intelligent VTC ligand design for future therapeutics.

## 1 Introduction

Particulate drug delivery was initially developed to package systemically toxic drugs into inert particle carriers, thereby selectively releasing active cargo to a diseased target. This approach should dramatically increase treatment efficacy by delivering more drug into the diseased tissue while eliminating systemic release, thereby mitigating toxic side effects. While this concept has not yielded a true “magic bullet” to date, packaging small molecule drugs into particles dramatically alters their pharmacokinetic/dynamic behavior and provides opportunities to direct drugs into diseased targets depending on their size, shape, and composition.^1^, ^2^, ^3^, ^4^ Many diseases manifest in the upregulation or overexpression of certain cellular surface receptors; therefore, tissue specificity can be enhanced with use of ligand‐receptor (LR) pairs. Particulate drug carriers can be coated with ligands complimentary to these receptors, providing a lock and key approach to disease‐specific delivery. In principle, the concept of LR pairs seems straightforward, however, implementation of actively targeted particles has proved challenging.^5^ Nanoparticle therapeutics with applications in cancer have driven research in the field. Despite conflicting results in overall success,^5^, ^6^, ^7^ a handful of candidates are currently in the clinical trial pipeline for cancer applications.^6^, ^8^

Vascular‐targeted carriers (VTCs) are an emerging area of research within particulate drug delivery. We define VTCs as particles designed with surface adhesive ligands that mimic those of leukocytes. During inflammation, activated endothelial cells (ECs) upregulate surface leukocyte adhesion molecules (LAMs), including selectins, intercellular adhesion molecule‐1 (ICAM), and vascular cell adhesion molecule‐1.^9^ Importantly, ECs shed their glycocalyx barrier, enabling leukocytes to interact directly with the EC surface.^10^ Surface ligands on circulating leukocytes facilitate rolling on and firm capture to activated ECs and assist in extravasation into tissue to perform various immune functions.^11^ This leukocyte adhesion cascade (LAC) is an important mechanism for normal immune function, but is also indicative of excessive cell recruitment that occurs early in many diseases.^9^, ^11^ Overexpression of LAMs represent potential targets for the design of particulate therapeutics; VTCs have been designed utilizing a wide range of LAM‐LR pairs for novel therapeutic approaches in atherosclerosis^12^, ^13^ and cancerous tumors,^14^ among others.^2^, ^6^, ^15^, ^16^, ^17^

Success of VTCs relies heavily on LR interactions with overexpressed LAMs on diseased ECs. VTCs are unique in that they must adhere to diseased ECs under rapid blood flow conditions, unlike other particles targeted to tissue spaces. Once localized to the endothelium, the interactions between LR pairs dictate adhesion and ultimate drug carrier efficacy. VTC and EC surface bonds must form rapidly to overcome particle momentum in flow. Capture and firm arrest at the surface will depend on the kinetics of the LR engaged pair and the aggregate strength of those interactions. Particle ligand total receptor avidity and specificity must be balanced; excessive avidity can lead to off target binding and immune responses due to rapid opsonization of non‐native proteins, while insufficient LR avidity can result in minimal binding.^18^ The design of VTC ligands must correspond with receptors in the targeted disease state; a ligand for the immediate onset of disease may not function efficiently in a chronic response.^19^, ^20^ Given the fluctuation of receptors on ECs and the presence of blood flow, leukocytes achieve adhesion with multiple LR pairs, where each LR pair provides a unique benefit of capture, firm adhesion, or transmigration, based on spatiotemporal expression on the diseased ECs. Notably, selectin receptors facilitate leukocyte capture and rolling, yet physiological levels of this LR pair are not enough to achieve firm adhesion on inflamed ECs. Firm adhesion requires secondary LR pairs, usually involving cell adhesion molecules (CAMs), which are expressed at a lower EC surface density with more favorable kinetics for firm adhesion.^9^, ^11^ Through the synergistic effects of these two LR pairs, leukocytes efficiently respond to inflammation on diseased tissues in vivo.

Despite the goal of LAC mimicry, the use of multiple LAM ligands on VTCs remains poorly understood. Most studies of VTCs have focused on particle designs with a single LR pair, with emphasis on the final disease outcome due to the delivered drug. Efficacy studies that have probed dual ligands are largely qualitative, comparing multiligand particles to the single ligand counterparts with minimal control over the total particle ligand presentation.^5^, ^18^, ^21^, ^22^, ^23^ Additional studies have probed the importance of ligand ratios between two LR pairs in static conditions, which fail to capture LR pair dynamics under physiologically relevant flows.^21^, ^24^ To study LR kinetics under flow, multiple research groups have used protein‐coated plates to study the adhesion and rolling of dual‐targeted particles in vitro.^25^, ^26^, ^27^, ^28^, ^29^, ^30^ However, these studies lack the complexity of a true diseased endothelium, as spatiotemporal LAM expression varies widely.^31^

To address these gaps in understanding, we have designed 500‐nm polystyrene spheres with controlled ligand densities and evaluated particle adhesion in physiological environments. Polystyrene particles serve as model VTCs in this work as it enables evaluation of ligand surface properties on a monodisperse particle population; we anticipate the particle dynamics observed here will be applicable to translatable particle formulations, such as poly(lactic‐*co*‐glycolic acid). While the 500nm size is just outside the 100–200nm range typically considered for drug delivery, 500‐nm spheres offer easier surface modification, characterization, and imaging, and have previously been shown to have similar blood flow adhesion dynamics as the 100–200nm spheres.^32^, ^33^ We investigated both single and dual LR pairs, exploiting selectin and intercellular adhesion molecule‐1 (ICAM) mediated paths of adhesion by designing particles with sialyl Lews A (sLe^A^) and anti‐ICAM1 (aICAM). The synergy between selectin:sLe^A^ (via PSGL‐1) and aICAM:β_2_‐integrin LR pairs drives optimal leukocyte adhesion during inflammatory events in vivo^9^; thus, representing a synergistic, leukocyte mimetic VTC system. Furthermore, the drastically different rates of interaction between the carbohydrate‐selectin LR pair and antibody‐CAM LR pair offers the opportunity for evaluating the role of LR pair kinetics in VTC design.^34^, ^35^ Notably, we evaluated these particles in a model of inflamed mesentery using intravital microscopy to capture a dynamic in vivo environment. Our results show that controlling the design of particle ligand presentation is critical in optimizing delivery of VTCs.

## 2 Materials and Methods

### 2.1 Study approvals

Human blood used in all assays was obtained via venipuncture according to a protocol approved by the University of Michigan Internal Review Board. Informed, written consent was obtained from all subjects prior to blood collection. Umbilical cords were obtained under a University of Michigan Medical School Internal Review Board (IRB‐MED) approved human tissue transfer protocol, which is exempt from informed consent per federal exemption category #4 of the 45 CFR 46.101.(b).

Animal studies were conducted in accordance with National Institutes of Health guidelines for the care and use of laboratory animals and approved by the Institutional Animal Care and Use Committee (IACUC) of University of Michigan. C57BL/6 mice were obtained from Jackson Laboratories. All animals were maintained in pathogen‐free facilities at the University of Michigan and used between 3 and 6 weeks in age.

### 2.2 Cell culture

Human umbilical vein endothelial cells (HUVECs) used in all assays were isolated from healthy umbilical cords (Mott Children's Hospital, Ann Arbor, MI) via a collagenase perfusion method.^36^ Isolated HUVECs were cultured in T75 flasks and seeded onto glass coverslips coated with gelatin (cross linked with glutaraldehyde) at 37°C and 5% CO_2_ with standard media until confluent density was reached.^31^

### 2.3 Flow cytometry of HUVECs

Six well plates were coated with gelatin (cross‐linked with glutaraldehyde) and seeded with HUVECs at a confluent density. The cells were activated with TNF‐α (Fitzgerald, North Acton, MA, 10 ng/mL in complete cell media) for varying time points. Following activation, the cells were trypsinized, divided into multiple samples, and stained with antibodies of CD54, CD62E, and an isotype control IgG1 (R&D Systems, Minneapolis, MN) at 4°C. All subsequent steps were performed at 4°C. After 20 min of staining, samples were washed twice in Phosphate‐Buffered Saline (PBS) with 0.5% BSA. Flow cytometry data were collected on an Attune NxT Focusing flow cytometer (Life Technologies, Waltham, MA) and analyzed using FlowJo software (Tree Star, Acton, OR). Data for activated cells are presented as compared to unactivated cells. All data have the appropriate isotype controls subtracted from the Median Fluorescent Intensity (MFI).

### 2.4 Particle functionalization

Carboxylated polystyrene (Fluoresbrite^®^ YG Polysciences, Inc., Warrington, PA) particles of 500nm diameter were covalently modified with NeutrAvidin^®^ Biotin‐Binding Protein (Thermo Scientific, Waltham, MA) via carbodiimide chemistry. Particles were washed with 2‐(N‐morpholino)ethanesulfonic acid (MES) buffer and incubated with a NeutrAvidin^®^ solution (5mg/mL) for 15 min at room temperature, after which an equal volume of *N*‐(3‐Dimethylaminopropyl)‐*N*′‐ethylcarbodiimide hydrochloride (EDC, 75mg/mL) was added and pH adjusted to 9.0. After incubating for 24 hr, glycine (7.5mg/mL) was added for 30 min to quench the reaction. NeutrAvidin^®^‐conjugated particles were then washed with a PBS buffer (50mM) and stored at 4°C until ligand conjugation.

For ligand conjugation, NeutrAvidin^®^‐conjugated particles were incubated for 45 min with a mixture of multivalent Sialyl Lewis^A^‐PAA‐biotin (sLe^A^, Glycotech, Gaithersburg, MD) and biotinylated antibodies (antimouse ICAM1, rat‐IgG2b, Biolegend, San Diego, CA, or antihuman ICAM1, R&D Systems) at room temperature. Following incubation, particles were washed with PBS buffer containing calcium and magnesium ions and 1% BSA and were then stored at 4°C until same day use for flow adhesion experiments, intravital microscopy, or ligand site characterization. Anticutaneous‐lymphocyte‐associated antigen‐APC (Miltenyi Biotech, Auburn, CA) and anti‐rat‐IgG2b‐PE (eBioscience, San Diego, CA) were used to calculate the corresponding ligand surface densities via flow cytometry as previously described.^37^, ^38^

### 2.5 Parallel plate flow chamber adhesion assay

Venous blood was collected from healthy adults into a syringe using acid‐sodium citrate‐dextrose (ACD) as anticoagulant and stored at 37°C until use; all assays utilized freshly drawn blood. ACD chelates calcium and inhibits particle internalization for the assay duration.^39^ Confluent HUVEC monolayers were activated with TNF‐α (Fitzgerald, 10 ng/mL in complete cell media) for 4 or 24 hr under static conditions at 37°C and 5% CO_2_ to induce E‐selectin and ICAM1 expression. Blood containing ligand‐coated particles at 5 × 10^6^ particles/mL was perfused over the activated HUVEC monolayer attached to a parallel plate flow chamber (PPFC, (PPFC, Glycotech, Gaithersburg, MD) Glycotech) in a laminar flow profile. The wall shear rate (WSR, γ_w_) was fixed to 200s^−1^ by adjustment of the volumetric flow rate (*Q*) through the channel, calculated by *Equation* (1),

(1) $$\gamma_{w}=\frac{6Q}{h^{2}w};s^{-1}$$

where *h* is the channel height (0.0127cm), *w* the channel width (0.25cm), and *Q* the volumetric flow rate (mL/s). *Q* was calculated as 82 µL/min in this system. The *h* of 127 µm and γ_w_ of 200s^−1^ were chosen to mimic the flow profile within a vein/venule of similar dimensions to those studied via intravital microscopy.^40^, ^41^ After blood perfusion of 5 min, PBS buffer containing 1% BSA was added to PPFC and particle adhesion densities were assessed via optical imaging using a Nikon TE‐2000‐S inverted microscope with a digital camera (Photometrics CoolSNAP EZ with a Sony CCD sensor). Due to the addition of buffer flow, only adherent particles are quantified in this assay. Results were imaged and analyzed via NIS‐Elements® analysis software and ImageJ.

### 2.6 Intravital fluorescence microscopy

Visualization of mesentery vessels was performed as previously described.^42^, ^43^ Briefly, female mice (3–4 weeks old) were anesthetized and a tail vein catheter placed for delivery of particles. Mice were placed on a custom‐made microscope heated stage at 37°C, and the mesentery was exteriorized to a glass cover slip via midline incision. Imaged vessels were chosen based on size, with the diameter of veins ranging from 100 to 200 µm, with an average of 153 µm. Following vessel selection, local injury was induced by topical application of TNF‐α (Fitzgerald, 10 µL of 200 µg/mL in PBS). Particles suspended in PBS were injected 3 min following topical TNF‐α application via IV catheter and imaged for another 5 min. Mice received 3 × 10^9^ particles in 200 µL injection volume, corresponding to ∼0.2mg/mouse, ∼10mg/kg. Targeted particle rolling and adhesion in mesenteric veins were visualised using a 25× oil objective on an inverted fluorescence microscope (Zeiss Axio Observer Z1 Marianas Microscope). Images were recorded continuously in green fluorescence every 10 ms using Slidebook 6 software.

Analysis was performed using Slidebook 6 and ImageJ using blinded file names. Particle rolling velocities were obtained using particle tracking software, and all paths were manually confirmed until at least 50 particles were tracked per experimental condition. Vessels were isolated and measured using Slidebook 6. Particles found in adjacent vessels but within the frame were excluded from the analysis. Particles were considered adherent when they appeared in the same location for 10 consecutive frames of the particle tracking. Particles were considered rolling when their tracked paths moved less than 50 µm between frames. Firmly adhered particles did not contribute to the rolling velocity data.

### 2.7 Particle adhesion simulation

Particle adhesion was simulated in a two‐dimensional rectangular channel using COMSOL 5.2 through a combined continuum and particulate model, adapted from previous work and described in detail in the Supporting Information Material.^30^, ^44^ Briefly, a velocity profile was established for an incompressible Newtonian fluid in a rectangular channel with dimensions 10 × 30 μm, with a reactive surface of 10 μm along the bottom wall. Unless otherwise designated, a WSR of 200s^−1^ was imposed (average channel velocity 1.67mm/s). A continuum model was developed to evaluate particle transport, considering both convection and diffusion and solved using the convection‐diffusion equation,

(2) $$\frac{\partial C}{\partial t}=D\nabla^{2}C$$

where *C* is the particle concentration and *D* is the particle mass diffusivity. The LR interaction at the reactive surface was treated with a general form boundary PDE, the governing equation being:

(3) $$\frac{\partial B}{\partial t}=k_{a}C_{w}-k_{d}B$$

where ***B*** is the number of bound particles on the reactive surface and *C*_w_ is the particle concentration near the wall. The variables *k*_a_ and *k*_d_ are the attachment and detachment rates of the ligand functionalized nanoparticles, respectively; both are functions of the forward (*k*_f_) and reverse (*k*_r_) LR bonding rates, the total number of ligands (*N*_L_) and receptors (*N*_R_), and the physical properties of the particles and fluid medium. These were approximated using a particulate model to capture the molecular level LR interactions, by establishing a total bond density, *N_b_*, between surfaces, with each LR interaction treated as independent values (*N_b_*_−1_ for ICAM‐aICAM interactions, *N_b−_*_2_ for selectin‐sLe^A^ interactions):

(4) $$N_{b}\left(t\right)=N_{b-1}+N_{b-2}\;=\;c_{17}+\frac{1}{c_{8}+c_{7}e^{-c_{6}t}}+\frac{1}{c_{16}+c_{15}e^{-c_{14}t}}$$

where *c*_7_, *c*_8_, *c*_17_, *c*_14_, *c*_15_, and *c*_16_ are constants containing *k*_f_, *k*_r_, *N*_L_‐sLe^A^, *N*_L_‐aICAM, *N*_R_‐selectin, and *N*_R_‐ICAM as derived in the Supporting Information Material. The forward (*k*_f_) and reverse (*k*_r_) LR bonding rates have been determined for aICAM/ICAM and sLe^A^/selectin in the literature, as reported in the Supporting Information.^34^, ^35^ Following analysis by Tan etal. and use of a force balance, representative times of *T*_r_, *T*_d_, and *T*_debond_ were determined using the expression of total *N_b_* to evaluate *k*_a_ and *k*_d_:

(5) $$k_{a}=\frac{d}{T_{d}+T_{r}}\;{\;k}_{d}\;=\;\frac{1}{T_{debond}}$$

where *d* is a representative length, chosen to be the diameter of the particle.^44^

Using the derived reaction boundary condition, ***B*** was determined as a function of time, *N*_L_‐aICAM, *N*_L_‐sLe^A^, *N*_R_‐ICAM, *N*_L_‐selectin, shear rate, and position on the reactive surface for a constant uniform inlet concentration of particles at 5 × 10^9^/mL. To obtain the total concentration bound, ***B*** was integrated over the 10‐µm reactive boundary. As this model does not incorporate the variable regio‐specific presentation of receptors at the endothelium wall, or differences between the interaction strengths of sLe^A^/selectin and aICAM/ICAM, the range of ligand and receptor densities of the four parameters were evaluated under conditions where each of these four parameters contributed to particle binding, as listed in the Supporting Information Material.

### 2.8 Statistics

Characterization of HUVEC expression after TNF‐α activation is representative of two independent experiments from different cell isolations, with two technical replicates each. PPFC flow experiment data is an average of 10 pictures from each individual experiment, with *n* ≥ 3 blood donors for each group of data presented. Intravital results represent averages from at least three different imaging sequences of different vessels within groups, *n* ≥ 3 mice per group. For all studies, all data points were included in the analyses and no outliers were excluded in calculations of means or statistical significance. Data are plotted with standard error bars and analyzed as indicated in figure legends. Asterisks indicate *p* values of *< .05, **< .01, ***<.001, and n.s indicates not significant.

## 3 Results

### 3.1 Density of sLe^A^ dictates particle adhesion in vitro and in vivo

**Figure 1:**
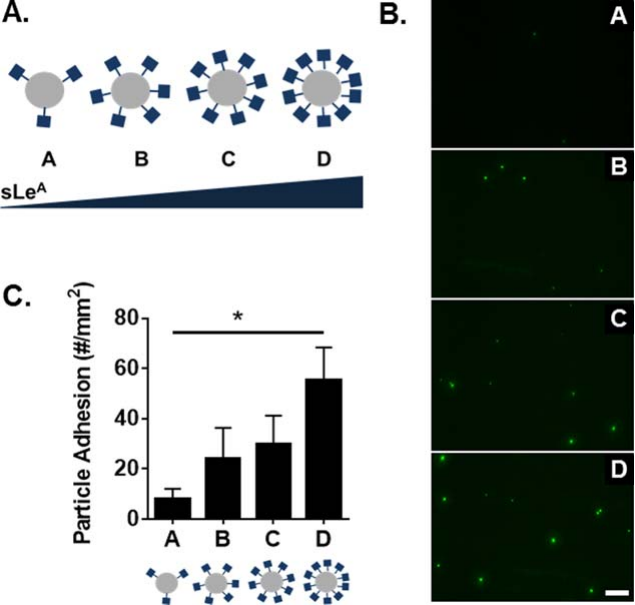
Particle adhesion to inflamed HUVEC monolayer as a function of total sLe^A^ sites. (A) Diagram of four particle conditions A–D with increasing sLe^A^ ligand density (5,000, 10,000, 20,000, and 40,000 sLe^A^ sites/µm^2^). (B) Representative fluorescence images of particle adhesion to in vitro inflamed HUVEC monolayer, corresponding to particles A–D from top to bottom. HUVEC activation was achieved via 4‐hr TNF‐α incubation. (C) Quantified particle adhesion. Statistical analysis was performed using one‐way ANOVA with Fisher's Least Significant Difference (LSD) test: (*) indicates *p* < .05, *n* = 3 donors. Error bars represent standard error, scale bar 50 µm

We utilized a PPFC assay to investigate the role of sLe^A^ density on particle adhesion under physiological blood flow conditions. A series of four particle types (A–D) was prepared with increasing sLe^A^ surface density (Figure 1A). SLe^A^ site densities were quantified by flow cytometry (Table 1), with representative gating shown in Supporting Information Figure 1 and reaction conditions in Supporting Information Figure 2. HUVEC monolayers were prepared and activated with TNF‐α for 4 hr prior to experiments. Representative fluorescent images of particle binding in the PPFC assay are shown in Figure 1B. Minimal nonspecific particle binding was observed for particles functionalized with an IgG‐isotype control of varied densities, as shown in Supporting Information Figure 3. Particle adhesion from sLe^A^ targeted particles was determined and nonspecific binding from control particles at corresponding site densities were subtracted out to quantify target‐specific adhesion (Figure 1C). Increasing the sLe^A^ density on 500‐nm particles from 5,000 to 40,000 sites/µm^2^ resulted in increased particle adhesion. We observed a 2‐fold increase in particle binding from A to B; additional sLe^A^ on C and D resulted in further increased adhesion (3‐ and 6‐fold increases over A, respectively).

**Table 1:** Particle ligand quantification^a^

Particle	sLe site density (#/µm^2^)	ICAM site density (#/µm^2^)	Total site density (#/µm^2^)
A	5,000	0	5,000
	5.642 ± 929	0	5.642
B	10,000	0	10,000
	10,374 ± 1,369	0	10,374
C	20,000	0	20,000
	19,623 ± 1.920	0	19,623
D	40,000	0	40,000
	41,040 ± 2,225	0	41,040
E	0	5,000	5,000
	0	4,526 ± 1,892	4,526
F	5,000	5,000	10,000
	3,866 ± 487	4,717 ± 1,006	8,583
G	0	10,000	10,000
	0	10,938 ± 2,536	10,938
H	10,000	10,000	20,000
	11,495 ± 4,631	10,987 ± 2,466	22,482
I	7,500	2,500	10,000
	6,792 ± 911	2,976 ± 675	9,768
J	2,500	7,500	10,000
	2,910 ± 127	8,891 ± 220	11,801

a Particle types A–J and corresponding ligand densities. Target values shown in gray, with actual values determined via flow cytometry shown below, *n* ≥ 2 particle batches, standard deviation shown. Representative gating is shown in Supporting Information Figure 1.

**Figure 2:**
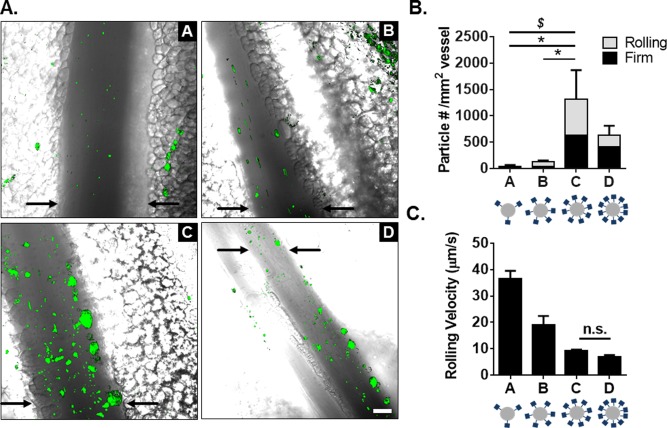
Particle adhesion to inflamed mesentery endothelium as a function of total sLe^A^ sites. (A) Representative fluorescence images of particle adhesion to inflamed mesentery, top images correspond to particles A and B (left to right, 5,000 and 10,000 sLe^A^ sites), bottom images correspond to particles C and D (left to right, 20,000 and 40,000 sLe^A^ sites). Particle fluorescence shown in green, overlaid on the bright field image. (B) Quantified adherent density of firmly bound and rolling particles per representative imaging segment, *n* = 3 mice. Statistical analysis was performed using one‐way ANOVA with Fisher's LSD test between total adherent particles: (*) indicates *p* < .05, and two‐way ANOVA with Fishers LSD test between groups: (*$*) indicates *p* < .05 between rolling groups, firm nonsignificant (n.s.). (C) Velocity of rolling particles found at mesentery wall, *n* ≥ 50 particles from *n* = 3 mice. Statistical analysis was performed using one‐way ANOVA with Fisher's LSD test, with all interactions *p* < .001 except where indicated. Error bars represent standard error, scale bar 50 µm

We were interested if the increase in sLe^A^ site density could produce a similar increase in particle binding in vivo, which we observed with real‐time intravital fluorescence microscopy. Fluorescent particle types A–D were visualized at the surface of the inflamed blood vessel in vivo, with qualitative differences in particle adhesion shown in representative still images of Figure 2A. The vessel walls of selected veins are indicated with black arrows; particles found in other vessels, including adjacent capillaries, were not included in the adhesion analysis. Particle adhesion and rolling densities were determined (Figure 2B) and the rolling velocities of corresponding particles are shown (Figure 2C). No particle binding or adhesion was observed for control particles with isotype‐control IgG (Supporting Information Video 1). Movies of particles B and C rolling in vivo can be seen in Supporting Information Videos 2 and 3.

Particles A–D successfully adhered to the inflamed endothelium via both rolling and firm arrest. Particle A exhibited the fastest rolling velocity, which corresponded to the lowest occurrence of particles firmly arrested or rolling at the wall. Particle B exhibited a decreased rolling velocity compared to particle A (*p* < .0001), which corresponded to an increased presence of particles at the wall. Particles C and D had similar low rolling velocities (*p* = .2), which resulted in more firmly bound particles; particle C was the most effective, with a ∼30‐fold increase of total adherent particles over A (*p* = .014). This corresponded to a ∼30‐fold increase in rolling particles and a ∼20‐fold increase in firmly arrested particles (*p* = .012 and *p* = .042, respectively). Additionally, particle C produced a ∼9‐fold increase in total adherent particles over B (*p* = .019), with a 7.5‐fold increase in number of rolling particles and a ∼10‐fold increase in firmly arrested particles (*p* = .019 and *p* = .051, respectively). We observed no statistical difference between particles C and D for either rolling or firmly arrested particles, however, the average number of adherent particles was less for particle D (*p* > .05 for all interactions). As the average velocity of rolling particles decreased, more particles of that type firmly adhered to the inflamed vessel. Particle types C and D had the highest sLe^A^ surface densities and yielded the highest amount of firmly arrested and rolling particles. This suggests a sLe^A^ saturation point in vivo.

### 3.2 Use of dual‐targeting ligands enhances particle binding in vivo

**Figure 3:**
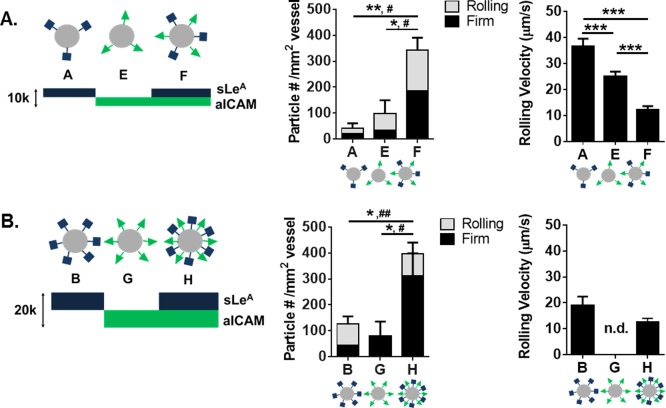
Particle adhesion to inflamed mesentery endothelium as a function of combined sLe^A^ and aICAM sites. Comparison of dual‐targeted particle designs with single ligand densities of (A) 5,000 sites/µm^2^ and (B) 10,000 sites/µm^2^. *Left*: Diagram of particle conditions with varied amounts of sLe^A^ and aICAM ligand density. *Middle*: Quantified adherent density of firmly bound and rolling particles per representative imaging segment, *n* = 3 mice. *Right:* Velocity of rolling particles found at mesentery wall, *n* ≥ 50 particles from *n* = 3 mice. Statistical analysis of adherent density was performed using one‐way ANOVA with Fisher's LSD test between total adherent particles: (*) indicates *p* < .05, (**) *p* < .01 and two‐way ANOVA with Fishers LSD test between groups, (#) indicates *p* < .05, (##) *p* < .01 between firm groups, rolling groups n.s. Statistical analysis of rolling velocity was performed using one‐way ANOVA with Fisher's LSD test, (***) indicates *p* < .001. Error bars represent standard error

We next explored how a mix of targeting ligands could further improve VTC adhesion in vivo. We compared a series of particles with varied sLe^A^ and anti‐ICAM (aICAM) ligand densities, as shown in Figure 3, to determine if particle adhesion from a dual‐targeted particle is merely the sum from the two individual ligands. The adhesion and rolling propensity of these particles were evaluated in the in vivo model of acute mesentery inflammation, as before.

As shown in Figure 3A, particles A and E had 5,000 sites/µm^2^ of sLe^A^ or aICAM, respectively, while particle F was the direct sum of the two ligands, for a total site density of 10,000 sites/µm^2^ (Table 1). At a constant ligand density of 5,000 sites/µm^2^, varying the ligand type from sLe^A^ (A) to aICAM (E) resulted in a statistically insignificant increase in particles rolling or firmly arrested on the vessel wall (*p* = .5). However, combining sites of sLe^A^ and aICAM on the same particle (F)resulted in a significant increase of adherent particles at the wall (∼7‐fold increase over A, *p* = .005, and ∼3‐fold increase over E, *p* = .012). For particle F, the number of firmly arrested particles was a ∼5‐fold (*p* = .034) and ∼3‐fold (*p* = .049) increase over A and E, respectively. No differences were observed in the number of rolling particles between groups, however, there were differences in rolling velocities. Particle A had the fastest rolling velocity, while particle F had the slowest rolling velocity, corresponding to the most effective adhesion at the wall. A video of particle type F adhering in vivo can be found in Supporting Information Video 4. With particle types A, E, and F, a decrease in observed particle rolling velocity corresponded to more particle adhesion at the vessel wall in vivo.

To probe the effect of total site density, we investigated particles B, G, and H, which each had twice the site densities of particles A, E, and F, respectively (Figure 3B). At these higher total site densities, varying the ligand from sLe^A^ (B) to aICAM (G) produced a minimal increase of particle presence at the wall, in either firmly arrested or rolling numbers, neither of which were significant from observation for particles A and E. Again, combining sites of sLe^A^ and aICAM on the same particle (H) resulted in a significant increase of adherent particles at the wall (∼2‐fold increase over B, *p* = .039, and ∼3‐fold increase over G, *p* = .030). The number of firmly arrested particles of type H resulted in a ∼4.3‐fold (*p* = .007) and ∼3‐fold (*p* = .011) increase over E and G, respectively. The relationship between rolling velocity and total particle adhesion was not linear in Figure 3B, owing to the fact that no particles were detected rolling for particle type G. Interestingly, increasing the total site density of dual‐targeted particles from 10,000 (F) to 20,000 (H) sites/µm^2^ did not provide a significant increase in adhesion (*p* = .5).

**Figure 4:**
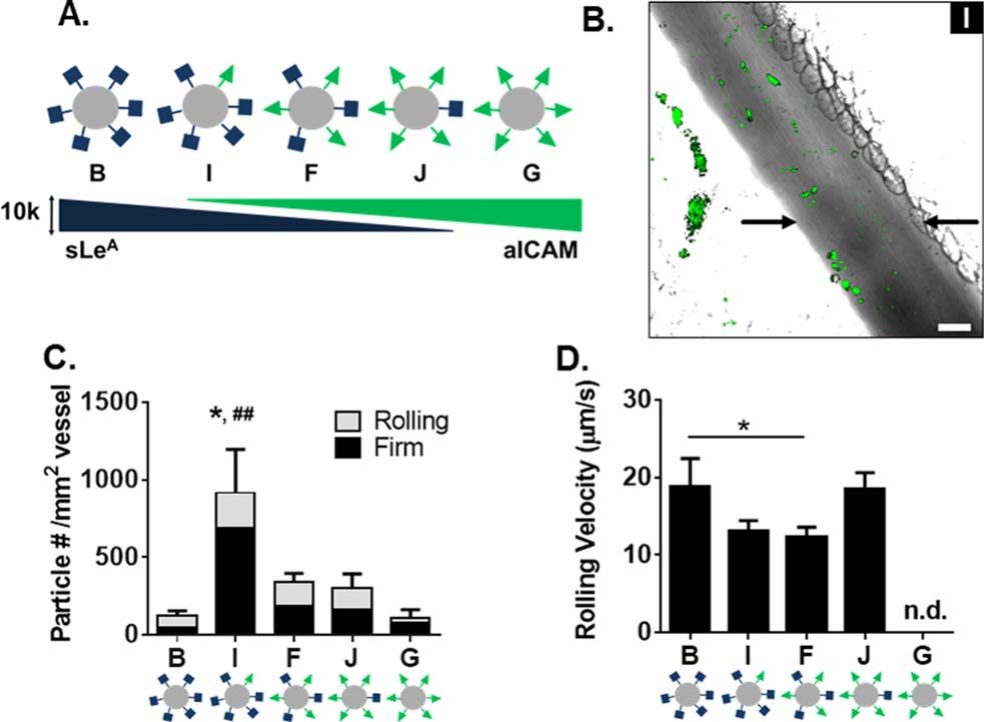
Particle adhesion to inflamed mesentery endothelium as a function of varied ratios of sLe^A^ and aICAM sites. (A) Diagram of five particle conditions with varied amounts of sLe^A^ and aICAM ligand density at a constant total site density of 10,000 sites/µm^2^. (B) Representative fluorescence image of particle I (7,500 sites/µm^2^ sLe^A^: 2,500 sites/µm^2^ anti‐ICAM) adhesion to inflamed mesentery, scale bar 50 µm. (C) Quantified number of fixed or rolling particles on inflamed mesentery per representative imaging segment, *n* = 3 mice. Statistical analysis of adherent density was performed using one‐way ANOVA with Fisher's LSD test between total adherent particles: (*) indicates *p* < .05 and two‐way ANOVA with Fishers LSD test between groups (##) *p* < .01 between firm groups, rolling groups n.s. (D) Velocity of rolling particles found at mesentery wall, *n* ≥ 50 particles from *n* = 3 mice. Statistical analysis of rolling velocity was performed using one‐way ANOVA with Fisher's LSD test, (*) indicates *p* < .05, n.d. indicates none detected and excluded from the analysis. Error bars represent standard error

Particle adhesion of both dual‐targeted particle types (F, H) indicates a more than additive effect of each individual‐targeting ligand. We further compared the benefit of dual‐targeting ligands on a single particle by keeping the total number of sites constant in order to eliminate any possible enhancement due to the change in total density (as studied in Figure 3). We developed a series of five particles with a constant total of 10,000 sites/µm^2^, given the lack of benefit when increasing to 20,000 sites/µm^2^ (F, H). We varied ratios of sLe^A^ and aICAM (Figure 4A and Table 1) and tested these in the model of acute mesentery inflammation. Figure 4B is a representative image of the highest dual‐targeted fluorescent particle binding in vivo. Representative movies of particle types F, I, and J can be found in Supporting Information Videos 4–6, respectively. For all five particle types, the number density of both rolling and firmly arrested particles is quantified in Figure 4C. The ligand combination on particle I resulted in a significantly increased number of firmly arrested particles compared to all other combinations (*p* < .001 for all comparisons), with at least a ∼3‐fold increase over all other ligand combinations. Similar numbers of rolling particles were observed with all five conditions; however, as shown in Figure 4D, slight decreases in rolling velocities were observed for particles I and F compared to B and J.

### 3.3 Optimal dual ligand ratio on particle varies with EC surface expression

**Figure 5:**
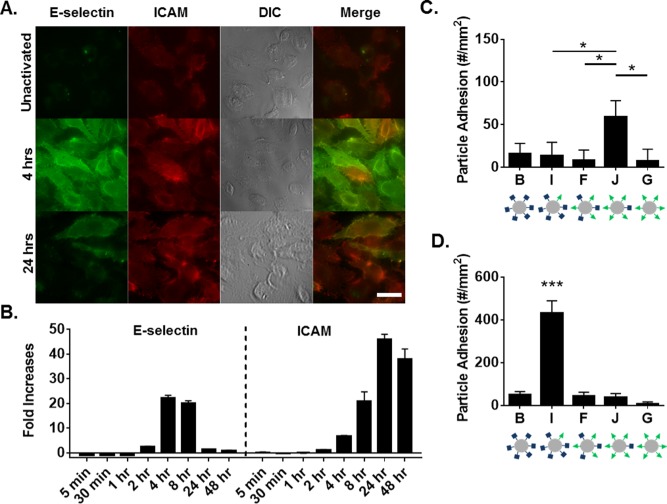
Particle adhesion to HUVEC monolayer as a function HUVEC surface expression. (A) Representative fluorescence images of HUVEC surface expression of E‐selectin and ICAM at 4‐ and 24‐hr post TNF‐α activation, scale bar 10 µm. (B) HUVEC surface expression of E‐selectin and ICAM (left to right) over time determined via flow cytometry, representative gating in Supporting Information Figure 5. Shown as fold increases of MFI over unactivated cells. Quantified particle adhesion following 5 min PPFC assay after (C) 4 hr and (D) 24 hr TNF‐α activation for particles at constant total site density but varied ligand ratios. Statistical analysis was performed using one‐way ANOVA with Fisher's LSD test: (*) indicates *p* < .05, (***) indicates *p* < .001 between all groups, *n* = 3 donors. Error bars represent standard error

Given the dramatic increase in adhesion of particle I in vivo over all other ligand combinations in the particle series (Figure 4A), we investigated the dependence of particle adhesion on the corresponding surface expression of LAMs on the endothelium. As shown in Figure 5A and Supporting Information Figure 4, TNF‐α activation of HUVECs resulted in elevated levels of both ICAM and E‐selectin surface expression, albeit maximally at different times. Basal levels of ICAM were observed in all three conditions, while no basal E‐selectin was observed. We further quantified these changes in expression level over time using flow cytometry (representative gating in Supporting Information Figure 5), with fold changes over unactivated cells shown in Figure 5B. Maximum E‐selectin was observed between 4 and 8 hr, while maximum ICAM expression was observed at 24 hr. With maximum E‐selectin expression, there was elevated ICAM expression, with the inverse true for E‐selectin at time points of maximum ICAM expression. We explored particle adhesion of the panel of five particle types from Figure 4A in a PPFC with activated HUVECs at 4 hr (Figure 5C) and 24 hr (Figure 5D) to probe the importance of HUVEC surface expression on particle adhesion. At 4 hr, particle J resulted in superior particle adhesion, corresponding to ∼1.5‐fold more bound particles than all other particle types (*p* < .05 between J and particles I, F, and G). At 24 hr, particle I resulted in maximal particle adhesion, with ∼6‐fold more bound particles over all other particle types (*p* < .0001 between all particle types).

**Figure 6:**
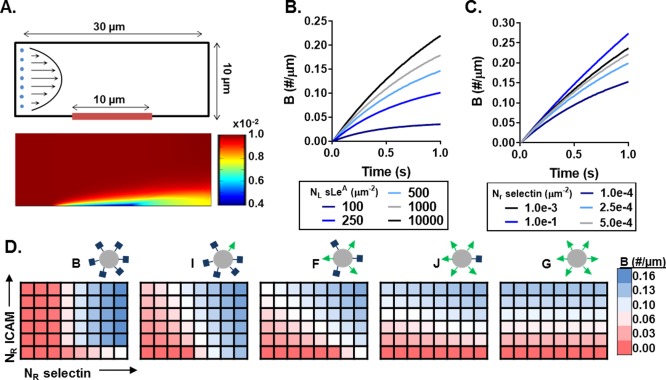
Computational model of particle adhesion as a function of ligand and receptor density. (A) Simulation geometry and flow profile (top), with representative resultant particle concentration profile within the fluid (bottom) in #/µm^2^. Bound particles not represented in the visualization. The bound particle concentration (***B***) over time at constant shear 200s^−^ 1 with (B) constant receptor (*N*_R_) density, and (C) constant ligand (*N*_L_) density. (D) Heat maps of B at 1s and constant shear 200s^−^ 1 as a function of *N*_R‐ICAM_ and *N*_R‐selectin_ for five particle combinations with varied *N*_L_ ratios. Blue indicates *N*_R_ conditions of more adherent particles and red indicating conditions of fewer adherent particles. *N*_R_‐ICAM ranges from 1 × 10^−6^ to 3 × 10^−4^/µm^2^, while *N*_R_‐selectin ranges from 1 × 10^−6^ to 3.5 × 10^−5^/µm^2^, both equally spaced

To further explore these LR‐pair interactions, we developed a computational model of binding at an endothelial surface, taking into account the number of ligands on the particle (*N*_L_‐aICAM, *N*_L_‐sLe^A^), the number of receptors on the endothelium (*N*_R_‐ICAM, *N*_R_‐selectin) and the dynamic properties of the particles under laminar flow. These variables were captured in the particle attachment (*k*_a_) and detachment (*k*_d_) rates at the boundary. The geometry of the 2D channel is shown in Figure 6A, which has a reactive region along the bottom of the surface; an example concentration profile within the fluid following the simulation is also shown. Adherent particles at the reactive surface are not visualized within the channel concentration profiles and are computed independently. Furthermore, only firmly bound particles are quantified at the surface; particle rolling was not incorporated in the model. From our derived expressions, we found that *k*_a_ and *k*_d_ depend dramatically on shear rate at constant ligand and receptor densities (Supporting Information Figure 6A). The *k*_a_ decreases slightly with increasing shear rate, while *k*_d_ increases over five orders of magnitude between tested shear rates of 10 and 1,000s^−1^. The dependency of *k*_a_ and *k*_d_ on shear rate translates to differences in particle adhesion at the surface, as shown in Supporting Information Figure 6A.

Using this model, we probed the differences in particle adhesion based on particle ligand ratios, for various surface receptor presentations of ICAM and selectin. To confirm that our model was sensitive to ligand and receptor densities of both LAM pairs, we independently varied *N*_L_ and *N*_R_ for both sLe^A^/selectin and aICAM/ICAM at a constant shear rate of 200s^−1^ (average channel velocity 1.67mm/s). Figure 6B shows the change in rate of bound particles (***B***) as a result of increasing *N*_L_‐sLe^A^, all else constant. This corresponds with results in Figure 1, confirming that the rate of ***B*** increases with increasing *N*_L_‐sLe^A^ density, but with diminishing returns. Similarly, Figure 6C shows as *N*_R_‐selectin increases, all else constant, the rate of ***B*** increases. These trends held true for aICAM/ICAM pairing as well (data not shown).

Having shown that our model could accurately capture the binding dynamics at the reactive surface for both LAMs, we compared the binding efficiencies of the particle series shown in Figure 4A over a range of *N*_R_ combinations. The heat maps in Figure 6D show the total number ***B*** after 1s for increasing *N*_R_‐selectin (*x* axis) and *N*_R_‐ICAM (*y*axis) expression, with blue and red indicating conditions of more and fewer adherent particles, respectively. Immediately, we observe distinct combinations of optimal binding for each particle type. Particle B, 100% sLe^A^, yields conditions with the largest magnitude of binding for the range of conditions modeled, yetalso yields negligible binding for over half of the conditions tested. Increasing the amount of aICAM on the particles while reducing the amount of sLe^A^ slowly shifts the conditions of favorable binding toward ICAM expression. Each of these five particle combinations with varied *N*_L_ yield unique binding profiles as a function of LAM surface expression (heat map differences between particles shown in Supporting Information Figure 6B). Figure 6 highlights the complex interplay between ligand and receptor densities combined with receptor‐ligand kinetics. For each particle type, endothelial surface expression prescribes its overall binding abilities. Thus, when designing targeted VTCs, it is key to understand endothelial surface expression patterns.

## 4 Discussion

Despite studies successfully employing dual‐targeted particles for vascular delivery, gaps still remain in understanding the effect of varied particle ligand densities and ratios. Here, we report a distinct interplay between endothelial receptor expression and particle ligand patterning that determines particle adhesion. To our knowledge, this is the first report of intravital microscopy investigations of dual‐targeting VTCs in vivo, thus allowing evaluation of both particle rolling and firm adhesion. We find that increasing the number of sites of sLe^A^ on particles increases adhesion in vitro and in vivo. We also find that the dual‐targeting particle designs result in adhesion superior to that of the linear addition of each individual ligand, indicating a multifaceted relationship in LR interactions. Overall, a 50%‐50% split in ligand coverage, which is often studied, did not result in the best adhesion tendencies in vitro or in vivo under the conditions explored; rather, intermediate ligand regimes produced the best performance. Our computational model supports this interplay between receptor density and dual‐targeted ligand ratios. Combined, these results indicate a balance required in LR kinetics, favoring particle designs with more ligands to the LAM receptor with the lower expression level.

Under flow conditions, many forces interact to prescribe the adhesion tendencies of VTCs. First, particles must marginate from bulk blood flow in order to interact with the vasculature of interest; only then does the targeting efficiency matter. Use of whole blood in our PPFC assay reproduces the conditions particles must overcome in vivo; particles must marginate to the surface, overcome collisions with blood cells, and be able to adhere in the presence of plasma proteins. Previous literature has demonstrated that 2–3 µm spherical particles are most efficient at marginating from bulk human blood flow and concentrate in the cell free layer near the wall, while 100–500nm particles remain uniformly distributed.^32^, ^33^ However, smaller nanosized VTCs remain appealing for the ability to safely traverse capillaries and travel through intracellular and interstitial spaces in both mouse and human circulation. As the particles used in this study were all of the same size and material, margination dynamics, and thus interaction potential with the vascular wall, can be considered uniform across all trials; all effects observed are attributable to variations in the surface ligand regimes.

In both our in vitro and in vivo results, increasing sLe^A^ densities produced increased adhesion. This increase in single ligand density also slowed the rolling velocity of particles, which has previously been demonstrated for sLe^A^ functionalized particles over a coated surface.^45^ As more LR pair interactions occur, the aggregate LR interaction forces overcome the wall shear force to establish firm adhesion. Avidin's multivalency increases the likelihood of clustered targeting ligands, yet our surface densities never saturated the available avidin sites (Supporting Information Figure 2). General increases in ligand density increases the likelihood of a viable spatial orientation that favors LR interactions. However, there were diminishing returns to adding more sLe^A^ on the particle surface in vivo. Other researchers have discussed the steric hindrance limit of antibody ligands, such that too many antibodies on a particle surface block each other from successful interaction with endothelial receptors.^46^, ^47^ Although sLe^A^ is a small carbohydrate unlikely to cause steric hindrance, our results indicate a similar trend, showing a clear limit of diminishing returns of particle adhesion with increasing site density. Adding more sites of sLe^A^ did not significantly hinder particle adhesion over the range of densities tested, but, these data disprove the mantra that “the more, the better” for particle ligands.

The dual‐ligand VTC particles studied here target both the selectin and β_2_ integrin mediated paths of adhesion to inflamed endothelium. Most previous work in dual ligand particles explores targeting inflammatory surface molecules with mixes of antibodies.^18^, ^21^, ^22^, ^48^ Our work aims to explore a dynamic mixing of a carbohydrate‐selectin LR pair with an antibody‐CAM LR pair, which have drastically different kinetics.^34^, ^35^ We first explored the benefit of having two ligands on a single particle, to determine if particle adhesion from a dual‐targeted particle is merely the sum from the two different ligands. To answer this, we evaluated the series of particles shown in Figure 3. In vivo, increasing the total ligand density of either sole ligand type from 5,000 to 10,000 sites/µm^2^ resulted in nonsignificant increases in either firmly arrested (A‐B, E‐G) or rolling (A‐B, E‐G) particles. When we compare these individual ligand types to dual‐targeted particles F and H, we can conclude that there is a more than additive effect when blending the two ligand types. For example, the total number of adhesive particles for dual‐targeted particle F is more than the addition of particle A and E; this is also true when comparing particle H to the sum of adhesion from B and G. Particles F and H resulted in increased rolling and adhesive densities compared to both of the single‐ligand particle types. As explained with the rolling velocities, this is likely due to the synergistic activity of the two ligands; sLe^A^ facilitates initial adhesion, but the rapid off rate guarantees some level of particle rolling,^34^ while aICAM facilitates firm adhesion after an initial interaction.^35^ When both ligands are present, the behaviors blend to allow initial rolling and eventual firm capture, similar to leukocytes. Various investigations into leukocyte adhesion in vivo have demonstrated the importance of endothelial expression of both selectins and CAMs on cell rolling and adhesion.^49^, ^50^, ^51^, ^52^ Providing a variety in the ligand presentation on particles corresponding to physiological ratios of endothelial receptors provides additive benefits for VTCs targeting a dynamic endothelium.

As Figures 1–2, and 6B demonstrate, increasing the total site density can increase particle adhesion both in vitro, in vivo, and in silico. To eliminate the suspicion that comparative observations in Figure 3 are purely due to the increased total site density, we compared particles of the same total ligand density (10,000 sites/µm^2^), as shown in Figures 4–6. Our results show that an optimal particle ligand ratio exists based on the surface expression of the endothelium, where the most effective particle type has a blend of both ligands and a ratio favoring the least expressed receptor. These results were initially counterintuitive. At maximum expression of E‐selectin (4 hr, Figure 5C), we hypothesized that the sLe^A^/E‐selectin interaction would control particle adhesion, with greater amounts of sLe^A^ resulting in greater adhesion to the dominant receptor. Instead, our results show the most adhesion for particle J, with 25% sLe^A^:75% aICAM. A parallel trend was observed at maximum expression of ICAM (24 hr, Figure 5D), where the most adhesion was achieved with particle I, with 75% sLe^A^:25% aICAM. Furthermore, the in vivo results shown in Figure 4C match this adhesion trend in Figure 5D. The receptor profile was not explicitly quantified in the mesentery, but it is known that rapid inflammation induced by topical TNF‐α results in P‐selectin expression within minutes of stimulation, in addition to omnipresent basal levels of ICAM.^53^, ^54^ SLe^A^ binds nonspecifically to all selectins, facilitating the adhesive sLe^A^‐selectin LR pair interaction. Although uncharacterized, the total number of ICAM receptors is likely higher than P‐selectin within our short, 3 min activation.^55^ Combined, these in vitro and in vivo data support the conclusion that the best ligand design of a particle is dependent on the surface expression of the ECs, showing better adhesion with more ligand for the lesser‐expressed receptor.

These results are further explained by the *in silico* model. While simplistic in particle dynamics in blood flow, this model crucially incorporates true kinetics of each LR pair, as well as the shear force dynamics of particles at the wall. The model provides a method to compare particle adhesion for a range of particle ligand combinations, incorporating LR‐pair kinetics. The heat maps in Figure 6D demonstrate how particle binding patterns shift with both selectin and ICAM receptor densities, across five particle types with a constant total number of sites. These diverse profiles indicate that each surface receptor presentation corresponds with an optimal particle‐ligand ratio, as driven by the kinetics of the LR pairs involved. There is a clear balance between ligand types based on differences in their on/off rate at the molecular level, which drives *k*_a_ and *k*_d_ at the transport continuum level. Furthermore, these heat maps corroborate the in vitro and in vivo trends of dual‐targeted particle adhesion. At the highest levels of selectin receptor and basal levels of ICAM in silico, particle I exhibits the highest binding of the five particles tested. The *in silico* model also identifies ranges of receptors which would corroborate the parallel trend at maximum ICAM. Such consideration of the LR pair kinetics and flow conditions explains our in vitro and in vivo findings of ligand preference to reach the less dominant receptor. Fewer ligands to the more abundant receptor are required to maximize the benefit of that LR pair (rolling or firm adhesion, for sLe^A^ and ICAM, respectively). Additionally, more ligands for the lesser‐expressed receptor increases the likelihood that the ligands find the LR pair for an adhesive interaction. Our *in silico* analysis provides clear validation that particle binding depends strongly on the LR kinetics and a balance of particle ligand and target receptor densities. This model could be readily applied to other combinations of LR pairs to predict particle adhesion; the corresponding receptor densities of a given surface would allow comparison between particle designs in order to determine the particle with the highest binding potential.

Few studies have delved deeply into the direct effect of each LR pair on multitargeted particles.^30^, ^35^, ^48^ Of those, there has been in vitro research corroborating that particle adhesion and rolling velocities depend on both the receptor density, as represented by coated plate coverage, and the particle ligand density.^35^, ^56^ In these coated plate studies, particles with increasing amounts of aICAM provided improved firm binding regardless of dominant plate receptor composition.^28^ Particles functionalized in these studies had ligand site densities typically less than 1,000 sites/µm^2^.^35^ Our in vitro findings further differ in three key ways, utilizing TNF‐α activated ECs rather than receptor coated plates, 500‐nm particles instead of 6 µm, and whole blood in place of buffer flow, all of which more closely capture the physiological dynamics at the surface of the vascular wall. Our in vitro findings are also supported by recent work with 2‐µm particles coated with variable ratios of the antibody ligands aICAM and anti‐E‐selectin, where optimal binding was achieved by a particle of 70:30 aICAM:anti‐E‐selectin ligand composition following 4‐hr HUVEC activation.^48^ Unfortunately, the total ligand site density was not evaluated in this study. Here, we attribute the success of this particle combination to the surface receptor expression, rather than the cited geometry of the flow channel. This work on larger, 2‐µm particles suggests that the same optimal presentation of ligands on the particle can be extended to particles of different sizes.

Optimization of VTC particle designs that utilize dual targeting is expected to provide improved delivery to the vascular wall. As demonstrated via intravital microscopy, the dual‐targeted VTCs studied here rapidly and efficiently adhere to the inflamed endothelium. Importantly, these combinations of ligands provided minimal off target adhesion, resulting in high targeting specificity. Our work suggests that cargo‐loaded VTCs with these ligand decorations can provide highly efficient binding to the vascular endothelium, especially when optimized toward the known receptor profiles of the target disease. In addition to this application toward drug delivery, there are possible diagnostic applications of this work. Particle adhesion with dual‐targeted particles could help determine the surface expression of diseased endothelium, providing a diagnostic tool to determine the stage of a disease with a simple IV injection of a blend of dual‐targeted particles.

## 5 Conclusions

Overall, the work presented here represents a truly novel demonstration of particle binding to an inflamed mesentery via intravital microscopy. We have shown that the adhesive abilities of 500nm particles, which are not preferentially excluded to the vascular wall from blood flow, can be significantly improved by targeting ligand design. While the total number of sites of a single ligand can drive particle adhesion to the endothelium, combining LR pairs from multiple LAM interactions provides a more powerful approach. These dual‐targeted ligand designs should be optimized based on the surface endothelium, with ligand coating densities favoring the less‐predominant adhesive receptor, as driven by the LR pair kinetics. The knowledge presented here about the importance of the LR pair matching will help in the intelligent particle ligand design for future applications in all diseases benefitting from VTCs, including atherosclerosis, cancer, inflammation, and many more.

## Supporting information

Additional Supporting Information can be found the online version of this article at the publisher's website.

Supporting Information

Supporting Information

Supporting Information

Supporting Information

Supporting Information

Supporting Information

Supporting Information
