# Supplementary material for: Evaluation of receptor‐ligand mechanisms of dual‐targeted particles to an inflamed endothelium
Source: Bioeng Transl Med. 2016 Jun 20;1(1):103–15. doi: 10.1002/btm2.10008 (PMC5217161; doi:10.1002/btm2.10008)
Supplement: Supplementary file 7 — Supporting Information [file BTM2-1-103-s007.pdf]

SUPPLEMENTAL INFORMATION

Evaluation of Receptor-Ligand Mechanisms of Dual-Targeted Particles to an Inflamed Endothelium

Catherine A. Fromen[1], Margaret B. Fish[1], Anthony Zimmerman[1], Adili Reheman[2], Michael Holinstat[2,3], Omolola Eniola-Adefeso[1] *

Included:

Supplemental Video Captions

Supplemental Methods

Supplemental Figures

Supplemental Videos

Representative intravital imaging of particle adhesion to inflamed mesentery. Local injury was induced by topical application of TNF-α. Particles suspended in PBS were injected 3 mins following topical TNF-α application via IV catheter and imaged for another 5 mins. Mice received $3x10^9$ particles in 200 µL injection volume, corresponding to ~0.2 mg/mouse, ~10mg/kg. A single major vessel is visible in the center of the frame within each supplemental video, with particle fluorescence visualized in green. Images were recorded continuously in green fluorescence every 10 ms.

1. Supplemental Video 1: Control IgG
2. Supplemental Video 2: Particle B
3. Supplemental Video 3: Particle C
4. Supplemental Video 4: Particle F
5. Supplemental Video 5: Particle I
6. Supplemental Video 6: Particle J

Supplemental Methods

COMSOL Computational Model

The ligand-receptor interaction was modeled using a commercial finite element analysis software package, COMSOL 5.2. The channel itself is defined as a 2D rectangular channel with dimensions 10 x 30 μm, with a reactive surface on the center 10 μm of the bottom wall. The rest of the bottom wall and the entire top wall are impermeable (represented by a no flux condition). A standard size floating triangle mesh was used inside the flow channel, while an extra fine mesh was used near the reactive surface. This was to improve resolution about the surface by better tracking small changes in the concentration gradient near the wall.

Incompressible, laminar flow in a Newtonian fluid was assumed and no-slip conditions were applied to all surfaces except the inlet, outlet and reactive surface. Time independent Navier-Stokes equations were solved in each element to determine flow characteristics of the system. A laminar inflow with average velocity (v) was established and held constant through the entire channel, defined by a constant shear rate (γ) at the wall:

$$\gamma = \frac{6v}{H} \tag{1}$$

where H is the height in the channel.

A continuum model was developed to evaluate particle transport. Both convection and diffusion were considered and solved for using the convection-diffusion equation,

$$\frac{DC}{Dt} = D\nabla^2 C \tag{2}$$

where C is the particle concentration and D is the particle mass diffusivity. An initial concentration C = 0 was used. The particle diffusivity, D, was calculated from the Stokes-Einstein equation:

$$D = \frac{k_b T}{3\pi\mu D_p} \tag{3}$$

This model assumes dilute species, meaning a volume fraction of less than 0.01. A constant, uniform concentration of 5×10^9 particles/mL was used as a boundary condition at the channel inlet. Influx from the particles detaching from the reactive surface was also considered within the continuum model, as determined by our boundary condition. The effects of blood cells on particle transport are likely significant but are not considered in this model.

The ligand-receptor interaction at the reactive surface was treated with a general form boundary PDE; the governing equation being:

$$\frac{\partial B}{\partial t} = k_a C_w - k_d B \tag{4}$$

And initial conditions of:

$$\left.\frac{\partial B}{\partial t}\right|_{t=0} = 0, \quad B(t=0) = 0 \tag{5}$$

where B is the bound particle density on the reactive surface and C_w is the particle concentration near the wall.

The variables k_a and k_d are the attachment and detachment rates of the ligand functionalized nanoparticles, respectively; both are functions of the forward (k_f) and reverse (k_r) ligand-receptor bonding rates, the total number of ligands (N_L) and receptors (N_R), and the physical properties of the particles and fluid medium. These were approximated using a particulate model to capture the molecular level ligand-receptor interactions, by establishing a total bond density, N_b, between surfaces, as described previously.[1] We treated each ligand-receptor interaction independently (N_{b-1} for ICAM-aICAM interactions, N_{b-2} for selectin-sLe^A interactions), such that:

$$N_b(t) = N_{b-1} + N_{b-2} \tag{6}$$

This allowed for relationships for each ligand-receptor pair as follows. The bond density for a single ligand-receptor pair can be described as:

$$\frac{\partial N_{b1}}{\partial t} = k_{f1}(N_{L1} - N_{b1})(N_{R1} - N_{b1}) - k_{r1}N_{b1} \tag{7}$$

which reduces to:

$$\frac{\partial N_{b1}}{\partial t} = c_1 + c_2 N_{b1} + k_{f1}N_{b1}^2 \tag{8}$$

with constants defined as:

$$c_1 = k_{f1}N_{L1}N_{R1} \tag{9}$$

$$c_4 = c_2 + c_3 - k_{r1} = -k_{f1}N_{L1} - k_{f1}N_{R1} - k_{r1} \tag{10}$$

This nonlinear, nonhomogeneous differential equation is in the form of an Riccati equation, defined by the form:

$$\frac{dx}{dt} = \mathrm{P}(t) + \mathrm{Q}(t)x + \mathrm{R}(t)x^2 \tag{11}$$

These Riccati equations have a solution in the form:

$$\frac{dx}{dt} = -\big(\mathrm{Q}(t) + 2y_1\mathrm{P}(t)\big)z - \mathrm{R}(t) \tag{12}$$

using the following transformation:

$$z = \frac{1}{x - y_1} \tag{13}$$

where y_1 is a known solution of equation 7. In this case, a solution for equation 8 when $\frac{\partial N_{b1}}{\partial t} = 0$ yields:

$$c_5 = y_1 = -\frac{1}{2k_{f1}}\left(c_2 \pm \sqrt{c_2^2 - 4c_1k_{f1}}\right) \tag{14}$$

Applying the transformation in equation 13, equation 8 becomes:

$$N_{b1} = c_5 + \frac{1}{z} \tag{15}$$

and the differential equation transforms to:

$$\frac{dN_{b1}}{dt} = -(c_2 + 2c_5k_{f1})z - k_{f1} = -zc_6 - k_{f1} \tag{16}$$

Applying the solution of equation 12, yields:

$$z = -\frac{k_{f1}}{c_6} + c_7e^{-c_6t} = c_8 + c_7e^{-c_6t} \tag{17}$$

Thus, from equation 13:

$$N_{b1} = c_5 + \frac{1}{c_8 + c_7e^{-c_6t}} \tag{16}$$

Applying the boundary conditions $N_{b1}|_{t=0} = 0$

$$c_7 = -\frac{1}{c_5} - c_8 \tag{17}$$

This analysis was repeated for N_{b2}, such that:

$$N_{b2} = c_{13} + \frac{1}{c_{16} + c_{15}e^{-c_{14}t}} \tag{18}$$

Thus, from equation 6, the total bond density becomes:

$$N_b(t) = N_{b-1} + N_{b-2} = c_{17} + \frac{1}{c_8 + c_7e^{-c_6t}} + \frac{1}{c_{16} + c_{15}e^{-c_{14}t}} \tag{19}$$

where $c_{17} = c_5 + c_{13}$

Following analysis by Tan *et al.*, representative time T_r was determined using the expression of total N_b and using a force balance between the bond strength and the drag experienced by the particle. The bond strength is estimated by:

$$F_{bond} = N_bf \tag{20}$$

where *f* is the force of a single bond. The drag experienced by the spherical particle is:

$$F_{drag} = C_d v + \frac{2\tau}{r_0} \tag{21}$$

where C_d is the drag coefficient, v is the velocity, r_0 is the radius of the contact area circle, and τ is the hydrodynamic torque, defined by:

$$\tau = 12\pi R^3 \mu\gamma \tag{22}$$

where μ is the fluid viscosity, R is the radius of the particle, and γ is the wall shear rate as in equation 1. When $F_{bond} < F_{drag}$, the particle does not adhere to the surface. When $F_{bond} > F_{drag}$, particle adhesion occurs. Thus, T_r is defined as the minimal reaction time required to form enough bonds such that $F_{bond} = F_{drag}$:

$$\frac{F_{drag}}{f} = c_{17} + \frac{1}{c_8 + c_7 e^{-c_6 T_r}} + \frac{1}{c_{16} + c_{15} e^{-c_{14} T_r}} \tag{23}$$

From equation 23, T_r was determined using the Goalseek function in Excel. Using this minimal reaction time, we define our overall attachment rate constant k_a as:

$$k_a = \frac{d}{T_d + T_r} \tag{24}$$

where T_d is a diffusion time included by Tan *et al.*, defined as :

$$T_d = \frac{2R^2}{D} \tag{25}$$

A similar analysis was followed to determine the overall detachment rate constant k_d. Detachment rate constants are determined such that:

$$\frac{\partial N_{b1}}{\partial t} = -k_{r1} N_{b1} \tag{26}$$

with solution of:

$$N_{b1} = c_{18} e^{-k_{r1} t} \tag{26}$$

Implementing the boundary conditions that $\frac{\partial N_{b1}}{\partial t} = 0$ at $t = 0, c_{18} = c_5$. Thus the overall bond density of detachment is:

$$N_b = N_{b1} + N_{b2} = c_5 e^{-k_{r1}t} + c_{13} e^{-k_{r2}t} \tag{26}$$

We plug this expression into equation 20 and follow a similar force balance as before. Here, when $F_{bond} < F_{drag}$, the particle detaches from the surface. Thus, T_{debond} is defined as the minimal time required to remove enough bonds such that $F_{bond} = F_{drag}$.

$$\frac{F_{drag}}{f} = c_5 e^{-k_{r1}T_{debond}} + c_{13} e^{-k_{r2}T_{debond}} \tag{27}$$

From equation 27, T_{debond} was determined using the Goalseek function in Excel. Using this minimal debond time, we define our overall detachment rate constant k_d as:

$$k_d = \frac{1}{T_{debond}} \tag{28}$$

List of constants

Term	Description	Value	Units
μ	Viscosity	1.00E-03	pa*s
l_d	Bond Length	2.00E-08	m
r_0	Radius of Circular Contact Area	9.798E-08	m
f	Single Bond Force	1.00E-11	N
d	Particle Diameter	5.00E-07	M
k_{f-1}	Forward Reaction Rate ICAM/aICAM	156,000	$M^{-1}s^{-1}$
k_{f-1}	*	1.295E-14	m^2s^{-1}
k_{r-1}	Reverse Reaction Rate ICAM/aICAM	1.13E-4	s^{-1}
k_{f-2}	Forward Reaction Rate selectin/sLe^A	27,000	$M^{-1}s^{-1}$
k_{f-2}	*	2.241E-15	m^2s^{-1}
k_{r-2}	Reverse Reaction Rate selectin/sLe^A	3	s^{-1}
R	Particle radius	500	Nm

*to convert k_f to units of surface area/s, an arbitrary length of l_d was assumed.

Supplemental Figures

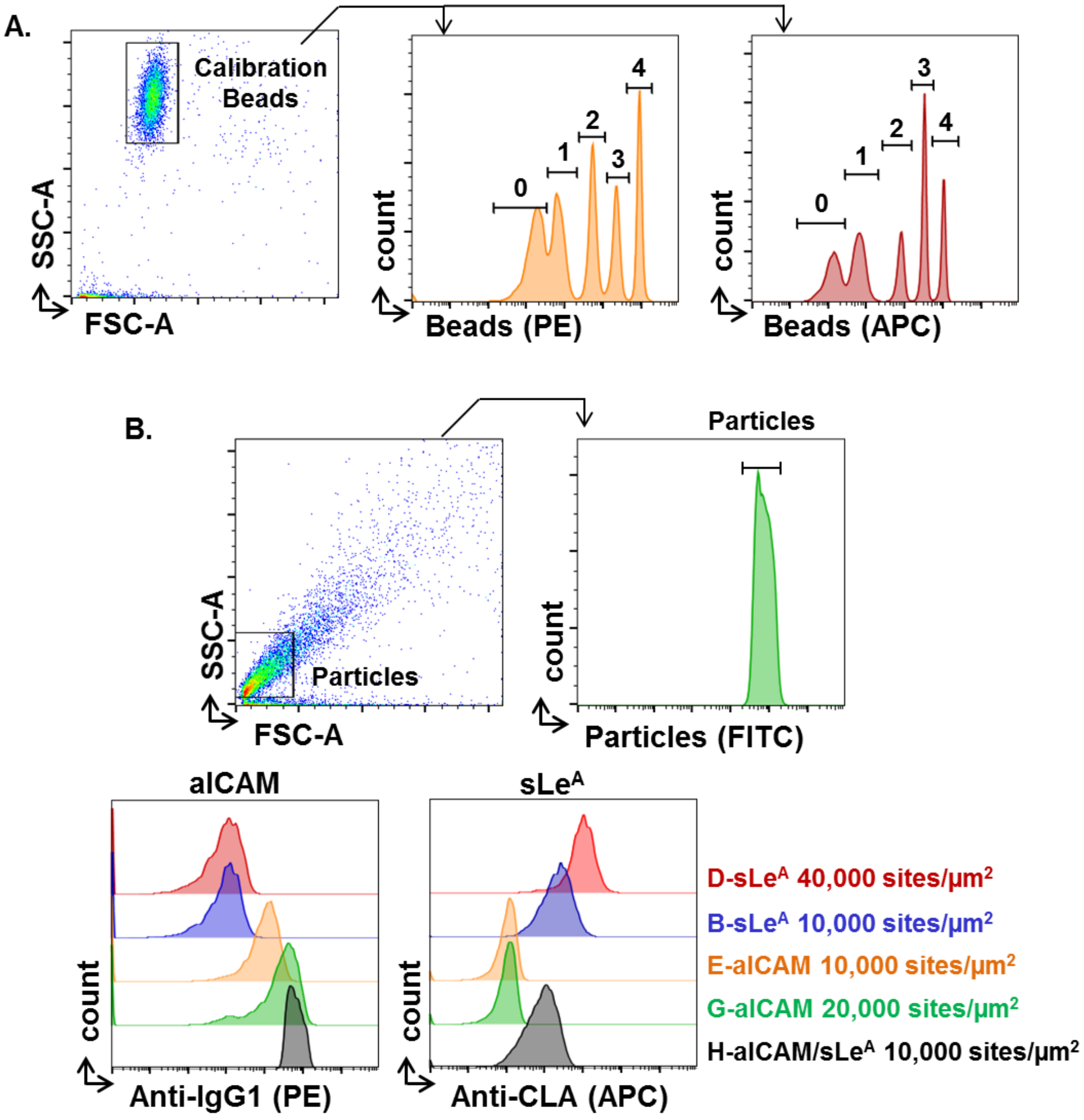

Supplemental Figure 1. Representative gating of particle site density calibrations. (A) Calibration beads from Bangs were run on the flow cytometer at the same voltages as the subsequent particles. The median fluorescence intensity (MFI) of each bead (0-4) was recorded for both PE and APC and a calibration curve made with known molecules of soluble fluorochrome (MESF) units provided by manufacturer. (B) Particles identified by forward (FSC) and side (SSC) area, and positive signal in the FITC channel (top). Representative histograms for a range of particles. MFIs for both PE and APC were determined for the corresponding antibodies indicating aICAM ligand (anti-IgG1) and sLe^{A} (anti-cutaneous lymphocyte associated antigen, CLA). MFIs were converted to units of MESF using the calibration curve from A.

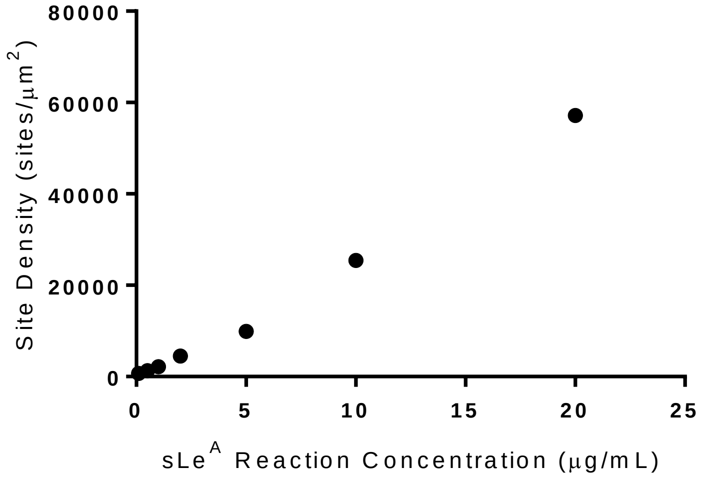

Supplemental Figure 2. Reaction conditions for biotinylated ligand attachment of sLe^A. Our highest site density used in subsequent studies was 40,000 ligands/μm^2; further increases in the ligand reaction concentration resulted in increased in ligand site density. This indicates that the active avidin sites were never saturated. At 40,000 sites/μm^2, the ligand occupies a parking area of 25 nm^2 per targeting ligand. This is five times larger than hydrodynamic radius of avidin (5.4 nm), thus implying a distributed ligand landscape.[2]

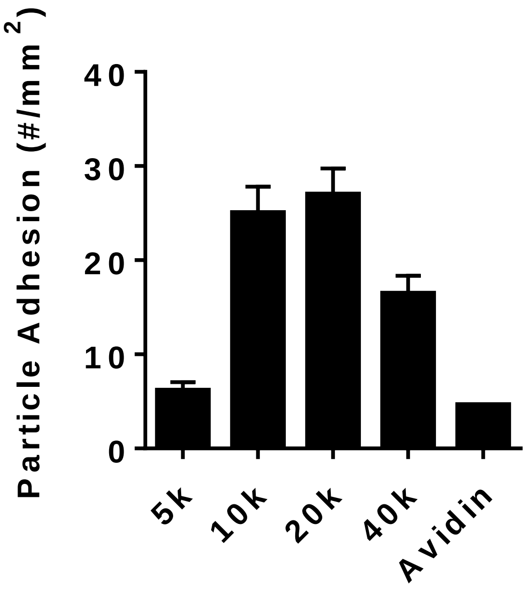

Supplemental Figure 3. Non-specific particle adhesion to inflamed HUVEC monolayer. Isotype-control IgG (biotin-rat IgG2b, Biolegend) sites were varied on particles ranging from 5,000, 10,000, 20,000, to 40,000. Using a PPFC, particle adhesion to *in vitro* inflamed HUVEC monolayer was determined HUVEC activation was achieved via 4 hr TNF-α incubation, n=3 donors. Error bars represent standard error.

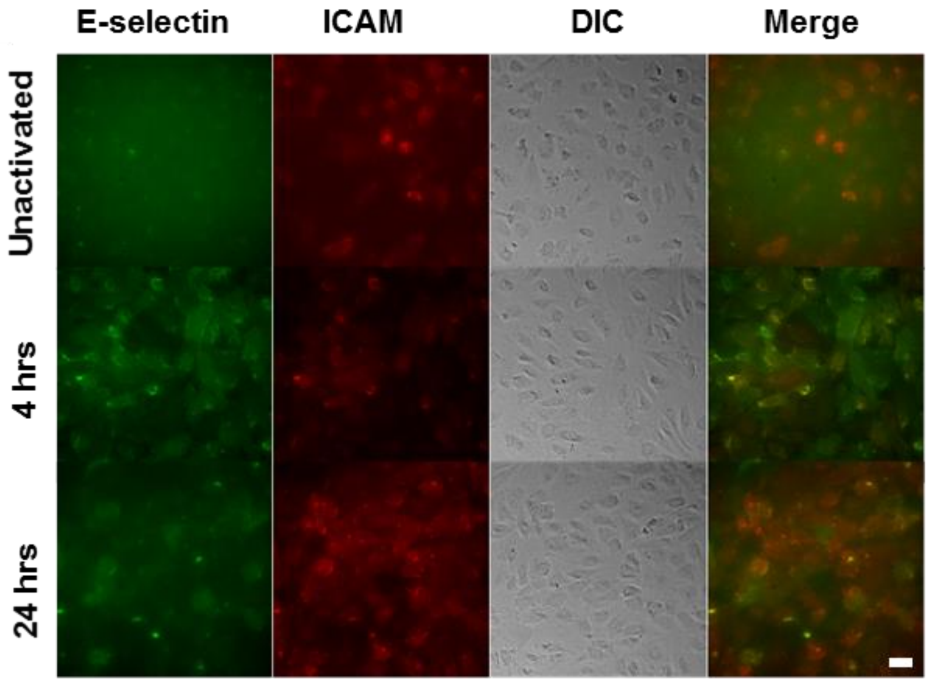

Supplemental Figure 4. HUVEC surface expression. Representative fluorescence images of HUVEC surface expression of E-selectin and ICAM at 4 hr and 24 hr post TNF-α activation. Scale bar 10 µm.

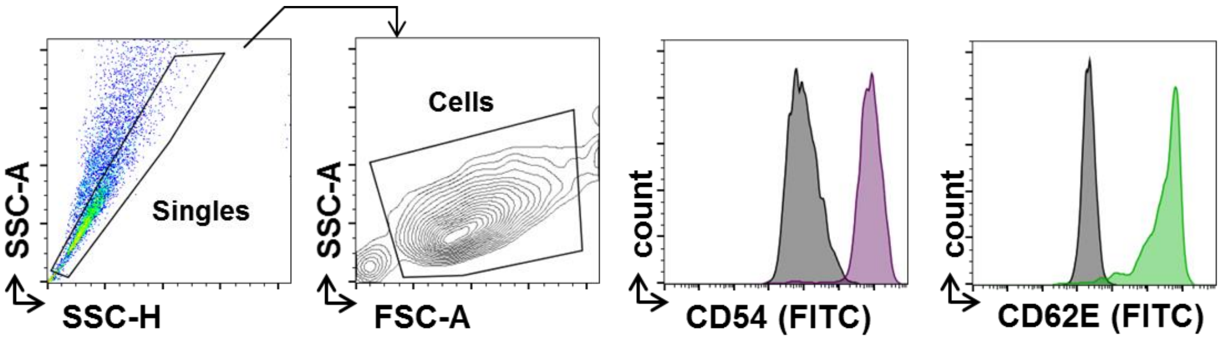

Supplemental Figure 5. Representative gating of HUVEC surface expression. HUVEC cells were identified as single cells by forward (FSC) and side (SSC). Surface expression of CD45 (ICAM) and CD62E (E-selectin) were evaluated by MFI values in the FITC channel. Untreated cells shown in black, purple curve for 24 hr TNF-α activation, green curve for 4 hr TNF-α activation.

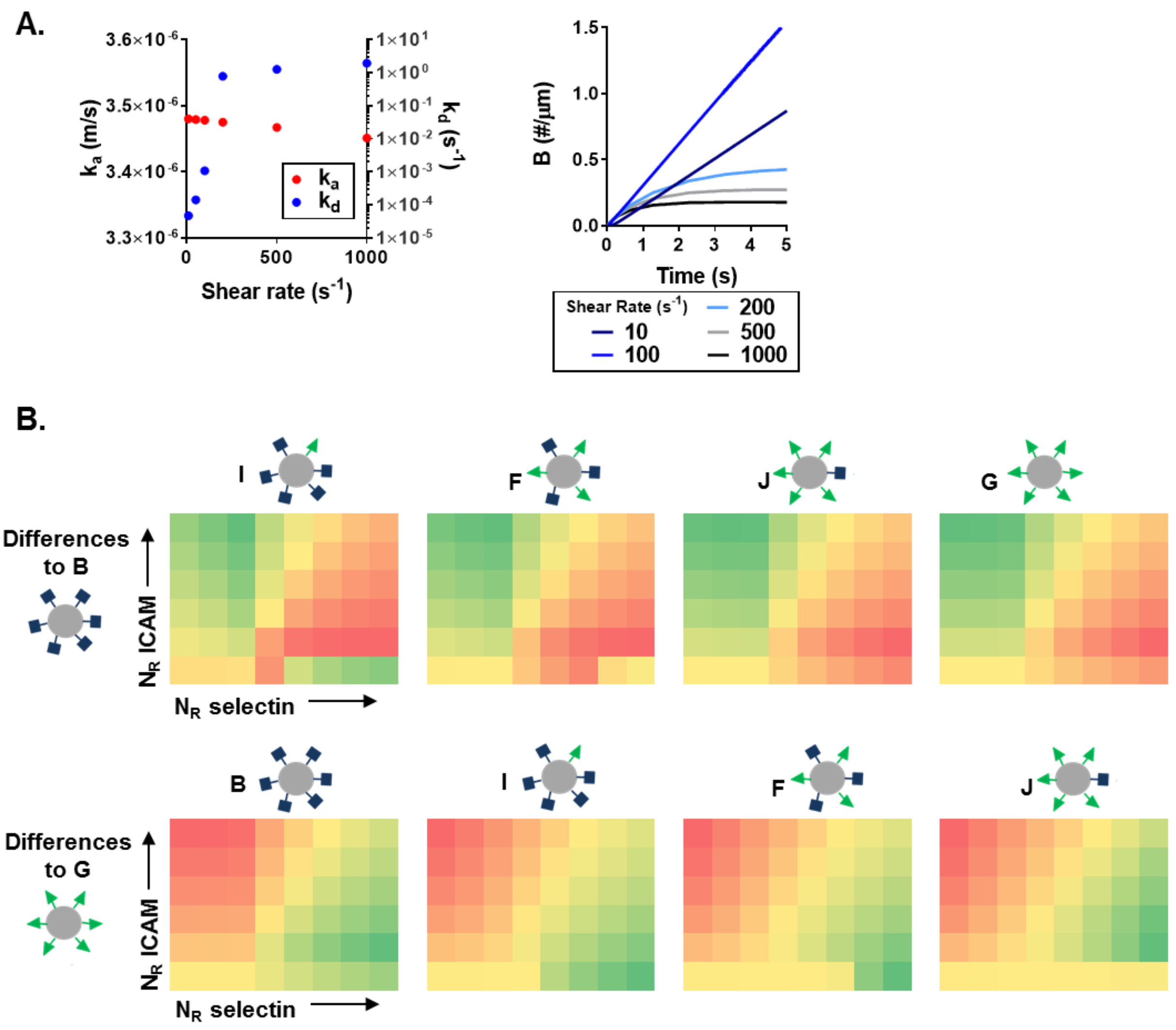

Supplemental Figure 6. Computational model of particle adhesion as a function of ligand and receptor density. (A) Attachment (k_a) and detachment (k_d) rate constants at varied shear rates determined from computational model and the corresponding bound particle concentration (B) over time at varied shear rates with constant ligand (N_L) and receptor (N_R) densities. (B) Differences in B between particle types determined at 1 sec and constant shear 200 s^{-1} as a function of $N_{R\text{-ICAM}}$ and $N_{R\text{-selectin}}$ for five particle combinations with varied N_L ratios. Green indicates N_R combinations with larger B than the comparison particle and red indicates N_R combinations with smaller B. N_R-ICAM ranges from to $1x10^{-6}$ to $3x10^{-4}$ μm^{-2}, while N_R-selectin ranges from to $1x10^{-6}$ to $3.5x10^{-5}$ μm^{-2}, both equally spaced.

References

1. Tan J, Wang S, Yang J, Liu Y. Coupled particulate and continuum model for nanoparticle targeted delivery. *Comput Struct*. 2013;122:128-134. doi:10.1016/j.compstruc.2012.12.019.
2. Liljeström V, Mikkilä J, Kostiainen M a. Self-assembly and modular functionalization of three-dimensional crystals from oppositely charged proteins. *Nat Commun*. 2014;5:4445. doi:10.1038/ncomms5445.
